# Eccrine Chromhidrosis and Bilioptysis in Type 1 Autoimmune Pancreatitis

**DOI:** 10.7759/cureus.73270

**Published:** 2024-11-08

**Authors:** Ahmed Alsaegh, Zainab Khamdan, Jalila Ahmed, Maryam Marhoon, Amina Ahmed, Adoub Alderazi, Hoor Husain

**Affiliations:** 1 Gastroenterology and Hepatology, Salmaniya Medical Complex, Manama, BHR; 2 Internal Medicine, Salmaniya Medical Complex, Manama, BHR; 3 General Medicine, Bahrain Ministry of Health, Manama, BHR; 4 Medicine, King Fahad University Hospital, Alkhobar, SAU; 5 Medicine, Salmaniya Medical Complex, Manama, BHR; 6 General Medicine, Alsalam Specialist Hospital, Manama, BHR

**Keywords:** autoimmune pancreatitis, bilioptysis, corticosteroids, eccrine chromhidrosis, jaundice

## Abstract

Autoimmune pancreatitis (AIP) is a rare form of pancreatitis. This case report focuses on type 1 autoimmune pancreatitis (AIP-1), an immunoglobulin G4 (IgG4)-related disease. It is characterized by dense infiltration of lymphocytes and plasma cells, primarily in a periductal distribution. We report a case of a 48-year-old man diagnosed with AIP-1 who subsequently developed eccrine chromhidrosis and bilioptysis during the peak of bilirubin level. This case highlights the diagnostic process followed to identify this challenging condition and demonstrates the patient’s response to the mainstay treatment. Moreover, it discusses the rare symptoms that can manifest with marked hyperbilirubinemia.

## Introduction

Autoimmune pancreatitis (AIP) is a rare variant of pancreatitis, defined clinically by the frequent presentation of obstructive jaundice, which may arise with or without a pancreatic mass. Histologically, it is characterized by the presence of lymphoplasmacytic infiltrate and fibrosis and, therapeutically, by a significant improvement with steroids. AIP has been classified into types 1 and 2 autoimmune pancreatitis (AIP-1 and AIP-2 [1,2].

AIP-1 is caused by IgG4 and usually presents with obstructive jaundice. It may occur alongside other IgG4-related conditions, such as sclerosing cholangitis, dacryosialadenitis, and retroperitoneal fibrosis, leading to the involvement of multiple organs. In contrast, AIP-2 is primarily idiopathic and confined to the pancreas, typically presenting with recurrent acute pancreatitis [1,2]. In most cases, AIP patients present with obstructive jaundice, epigastric pain, new-onset or worsening diabetes, and general fatigue [2]. Overall, the symptoms of AIP resemble those of pancreatic cancer, which can lead to overlapping diagnoses [1,2].

## Case presentation

**Figure 1:**
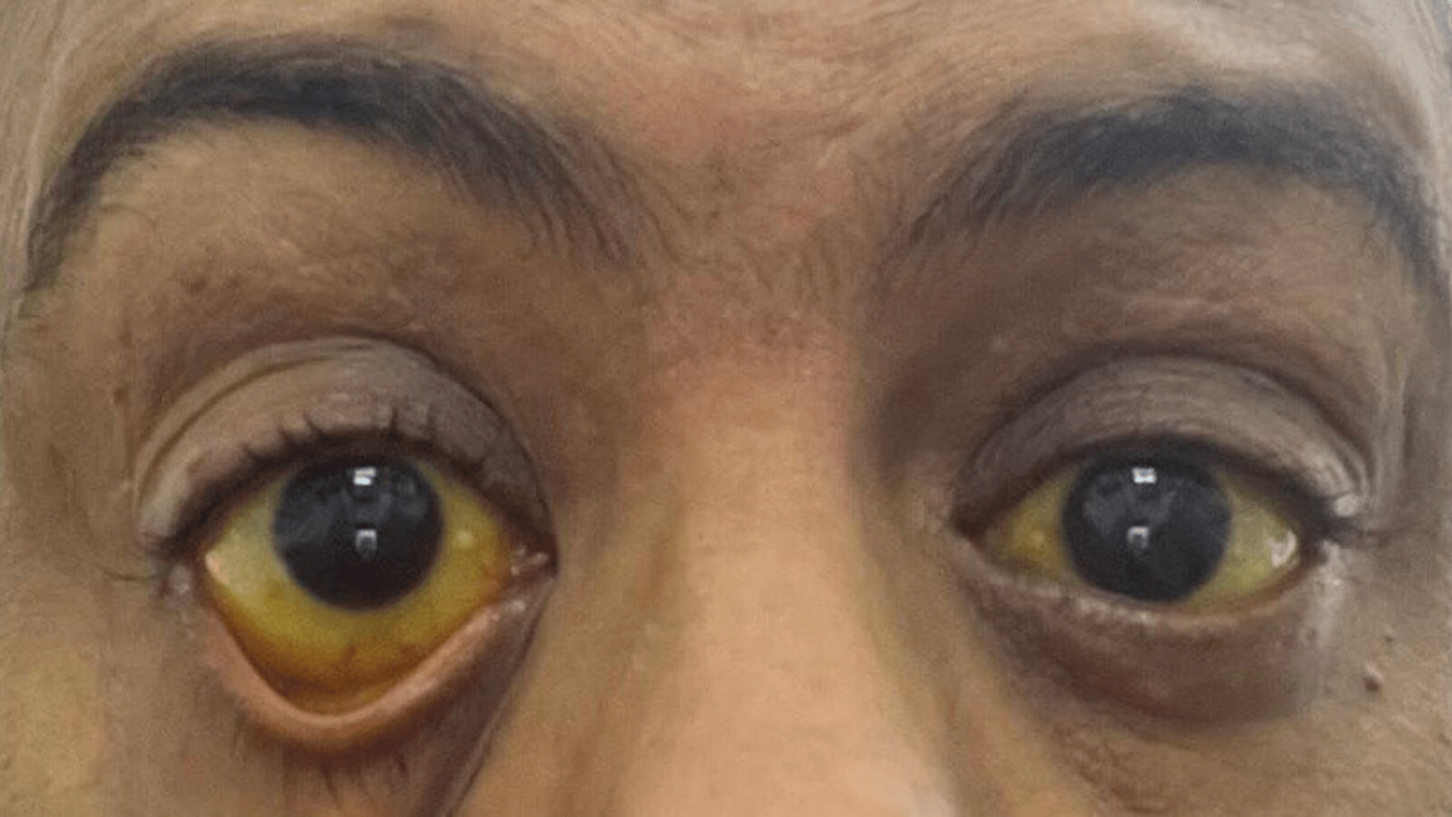
Patient’s eyes demonstrating jaundice upon initial presentation

The patient is a 48-year-old male with no significant medical history who presented to the emergency department with yellowish discoloration of the skin and sclera for three days (Figure 1). This was accompanied by loose, clay-colored stools and urine ranging from very dark yellow to brown. He experienced dull, intermittent epigastric abdominal pain. He reported easy fatigability, polyuria, polydipsia, and occasional pruritus. 

**Figure 2:**
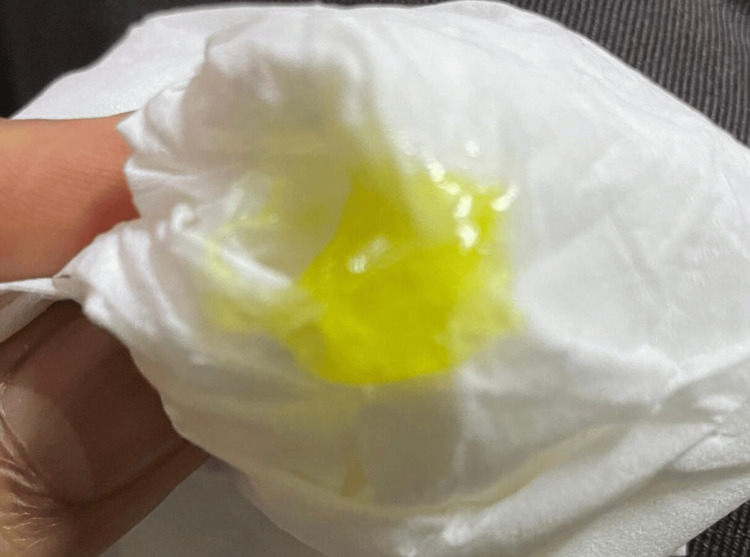
Expectorated sputum from the patient during the peak of bilirubin levels

On examination, the patient manifested jaundice in the form of pronounced yellowing of the skin and sclera, moderate abdominal tenderness, and no other significant findings. Laboratory results demonstrated a high total bilirubin of 137 µmol/L, which exhibited a continuing upward trend, reaching a maximum level of 490 µmol/L, predominantly involving the direct bilirubin fraction. At this stage, the patient reported having yellow sweat and producing bright yellow sputum (Figure 2) without signs and symptoms suggesting respiratory involvement. Additionally, liver enzymes were elevated, and fasting blood glucose and hemoglobin A1c were high, indicating the presence of undiagnosed diabetes mellitus. However, the amylase level was within the normal range (Table 1).

**Table 1:** Laboratory investigation results at initial presentation

Variable	Value	Reference Range
Liver function test		
Total bilirubin	137 µmol/L	5–21 µmol/L
Direct bilirubin	98 µmol/L	<5.1 µmol/L
Indirect bilirubin	39 µmol/L	<18 µmol/L
Alanine transaminase (ALT)	486 U/L	< 41 U/L
Alkaline phosphatase	231 U/L	46–116 U/L
G-glutamyl transferase	831 U/L	<73 U/L
Pancreatic enzymes		
Amylase	32 U/L	40–140 U/L
Diabetic profile		
Fasting blood glucose	11.8 mmol/L	Diabetic >7 mmol/L
Hemoglobin A1c	53 mmol/mol	Diabetic >47 mmol/mol

**Figure 3:**
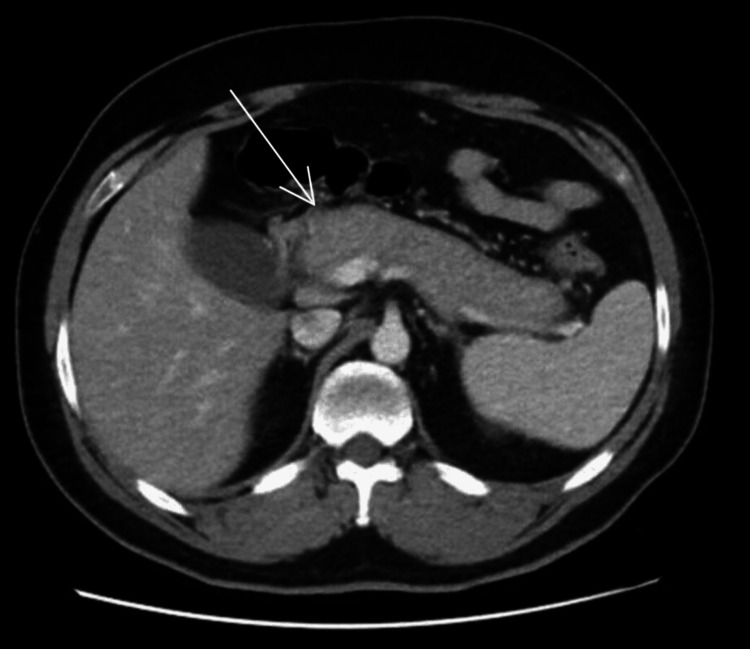
CT scan showing diffusely enlarged pancreas

Imaging, including a computerized tomography (CT) scan, revealed significant diffuse enlargement and edema of the pancreas with sub-centimetric prominent peripancreatic lymph nodes, suggesting pancreatitis (Figure 3). Additionally, the common bile duct (CBD) was dilated, measuring 1 cm in diameter. Subsequently, an IgG4 test showed a result of 138 mg/dL (normal level <135 mg/dL). An endoscopic ultrasound (EUS), fine needle biopsy (FNB), and ampullary biopsy were performed. EUS demonstrated a significantly enlarged pancreas with heterogenous lobulation of the parenchyma in the head and body, with hyperechoic foci; the main pancreatic duct was not dilated and had a hyperechoic wall. FNB histology revealed active fibroblasts without plasma cell infiltration or cytological evidence of malignancy, and IgG4-positive cell levels were noncontributory. Moreover, an ampullary biopsy showed moderate mixed inflammation and granulation tissue in lamina propria, associated with infiltration by plasma cells, lymphocytes, eosinophils, and occasional neutrophils, with no features of malignancy.

A course of prednisolone was commenced, starting at 40 mg daily for two weeks, followed by a tapering regimen of 5 mg per two weeks to a maintenance dose of 5 mg daily. The patient showed a significant response, manifested by the resolution of jaundice and a marked drop in bilirubin levels from 490 µmol/L to 143 µmol/L within two weeks. A follow-up CT scan showed a normal-sized, uniformly enhancing pancreas with no significant peripancreatic fat stranding or lymph nodes and a regression of the previously noted CBD dilatation. This significant improvement has added additional confirmation to the diagnosis.

## Discussion

The diagnosis of AIP-1 requires careful consideration of multiple clinical features and diagnostic criteria. As outlined by the International Consensus Diagnostic Criteria (ICDC) and Japanese Consensus Guidelines, the diagnosis of AIP-1 encompasses the following cardinal features: parenchymal imaging findings, assessment of pancreatic ducts via endoscopic retrograde pancreatography (ERP), IgG4 serology, other organ involvement, core biopsy histological results, and response to steroids. Although histological biopsy is useful in diagnosing AIP, it is not always necessary to establish a diagnosis of AIP-1. However, it remains essential for ruling out pancreatic cancer [1,2].

In applying the ICDC and Japanese Consensus Guideline, our patient has a definitive diagnosis of AIP-1, demonstrated by a diffuse pancreatic enlargement on a CT scan and an IgG4 level of more than 135 mg/dL. In addition, there was marked improvement with steroid therapy [1,2]. Despite inconclusive FNB results, an ampullary biopsy showing lymphoplasmacytic infiltration can support the diagnosis [2].

The presentation of yellow sweat (eccrine chromhidrosis) and yellow sputum (bilioptysis) observed in our patient has not been previously documented in the context of AIP in the medical literature. Eccrine chromhidrosis is a rare dermatological manifestation that develops from the excretion of water-soluble dyes or pigments from eccrine sweat glands. When hyperbilirubinemia is significant, the excreted bilirubin can result in yellow sweat. Park et al. reported nine cases of eccrine chromhidrosis associated with significant hyperbilirubinemia from various causes affecting the hepatobiliary system, none of which were AIP [3]. While bilioptysis refers to the presence of bilirubin in the sputum, it is typically linked to broncho-biliary fistulas [4]. There have been four reported cases of bilioptysis in conjunction with hyperbilirubinemia (Table 2). With total bilirubin ranging from 144 to 457.7 µmol/L. These cases involved underlying liver dysfunction or a hemolytic sickle cell crisis [4-7]. We suggest that the occurrence of these symptoms in our patient could be attributable to elevated bilirubin levels, particularly as they appeared when the bilirubin level peaked at 490 µmol/L.

**Table 2:** Our patient compared to prior cases of bilioptysis in the setting of hyperbilirubinemia Patients 1-4 were summarized previously. *Our patient had the highest bilirubin level at the time of occurrence.

Patient number	Age (years)	Sex	Pathology	Total bilirubin µmol/L
1 [4]	36	Female	Alcohol hepatitis	175
2 [5]	24	Male	Sickle cell disease	257.9
3 [6]	47	Male	Fulminant liver failure	457.7
4 [7]	45	Male	Alcoholic liver cirrhosis	144
5^*^	48	Male	Autoimmune pancreatitis	490

## Conclusions

In conclusion, this case report highlights a rare presentation of AIP-1 in a 48-year-old male, characterized by obstructive jaundice and distinct clinical symptoms, including eccrine chromhidrosis and bilioptysis, which have not been previously documented in the context of AIP. The diagnosis was supported by imaging findings, elevated IgG4 levels, and a dramatic response to steroid therapy, reinforcing the significance of recognizing AIP as a differential diagnosis in patients with obstructive jaundice. This case illustrates the importance of a thorough diagnostic approach, utilizing imaging and serological markers, while also highlighting the necessity of histological evaluation to exclude malignancy. The unusual symptoms seen in this patient highlight the varied clinical manifestations that can arise from significant hyperbilirubinemia. Further research can be conducted to study the prevalence of these symptoms in connection with hepatobiliary diseases and hemolytic conditions.
